# Structural and Functional Brain Network Connectivity at Different King's Stages in Patients With Amyotrophic Lateral Sclerosis

**DOI:** 10.1212/WNL.0000000000207946

**Published:** 2023-12-14

**Authors:** Edoardo G. Spinelli, Alma Ghirelli, Silvia Basaia, Elisa Canu, Veronica Castelnovo, Camilla Cividini, Tommaso Russo, Paride Schito, Yuri M. Falzone, Nilo Riva, Massimo Filippi, Federica Agosta

**Affiliations:** From the Neuroimaging Research Unit (E.G.S., A.G., S.B., E.C., V.C., C.C., M.F., F.A.), Division of Neuroscience, and Neurology Unit (E.G.S., A.G., T.R., P.S., Y.M.F., M.F., F.A.), IRCCS San Raffaele Scientific Institute; Vita-Salute San Raffaele University (E.G.S., A.G., T.R., M.F., F.A.); Neurorehabilitation Unit (N.R., M.F.), and Neurophysiology Service (M.F.), IRCCS San Raffaele Scientific Institute, Milan, Italy.

## Abstract

**Background and Objectives:**

There is currently no validated disease-stage biomarker for amyotrophic lateral sclerosis (ALS). The identification of quantitative and reproducible markers of disease stratification in ALS is fundamental for study design definition and inclusion of homogenous patient cohorts into clinical trials. Our aim was to assess the rearrangements of structural and functional brain connectivity underlying the clinical stages of ALS, to suggest objective, reproducible measures provided by MRI connectomics mirroring disease staging.

**Methods:**

In this observational study, patients with ALS and healthy controls (HCs) underwent clinical evaluation and brain MRI on a 3T scanner. Patients were classified into 4 groups, according to the King's staging system. Structural and functional brain connectivity matrices were obtained using diffusion tensor and resting-state fMRI data, respectively. Whole-brain network-based statistics (NBS) analysis and comparisons of intraregional and inter-regional connectivity values using analysis of covariance models were performed between groups. Correlations between MRI and clinical/cognitive measures were tested using Pearson coefficient.

**Results:**

One hundred four patients with ALS and 61 age-matched and sex-matched HCs were included. NBS and regional connectivity analyses demonstrated a progressive decrease of intranetwork and internetwork structural connectivity of sensorimotor regions at increasing ALS stages in our cohort, compared with HCs. By contrast, functional connectivity showed divergent patterns between King's stages 3 (increase in basal ganglia and temporal circuits [*p* = 0.04 and *p* = 0.05, respectively]) and 4 (frontotemporal decrease [*p* = 0.03]), suggesting a complex interplay between opposite phenomena in late stages of the disease. Intraregional sensorimotor structural connectivity was correlated with ALS Functional Rating Scale-revised (ALSFRS-r) score (*r* = 0.31, *p* < 0.001) and upper motor neuron burden (*r* = −0.25, *p* = 0.01). Inter-regional frontal-sensorimotor structural connectivity was also correlated with ALSFRS-r (*r* = 0.24, *p* = 0.02). No correlations with cognitive measures were found.

**Discussion:**

MRI of the brain allows to demonstrate and quantify increasing disruption of structural connectivity involving the sensorimotor networks in ALS, mirroring disease stages. Frontotemporal functional disconnection seems to characterize only advanced disease phases. Our findings support the utility of MRI connectomics to stratify patients and stage brain pathology in ALS in a reproducible way, which may mirror clinical progression.

## Introduction

Amyotrophic lateral sclerosis (ALS) is a fatal neurodegenerative condition that leads to degeneration of upper motor neuron (UMN) and lower motor neuron. It presents as a progressive loss of motor function, which ultimately causes death due to the involvement of respiratory muscles.^1^ Notwithstanding the great effort of the scientific community in the past decades, the impact of currently approved treatment for this condition for improved survival and quality of life is still marginal.^2^ Part of this issue could be possibly explained by the lack of disease-stage biomarkers, which hampers precise enrollment in clinical trials.

Accurate staging of patients with ALS is of paramount importance to allow a correct stratification of populations included into current and upcoming clinical trials, which need to be effectively designed to identify disease modifiers that could treat this condition. The ALS Functional Rating Scale^3^ has been widely used as a continuous variable describing the progression of functional impairment in this disease, although this measure alone is dependent on patient and caregiver's reports and does not provide a discrete classification into homogeneous subgroups of patients.^4^ King's staging is a simple, although schematic way to stage patients with ALS according to the number of body regions involved (stages 1–3) or the presence of respiratory/nutritional failure (stage 4).^5^ In the past decades, an effort has been made to identify objective, reproducible measures of CNS damage in ALS.^6,7^ In this context, neuroimaging has played a fundamental role.^6,8^ Advanced MRI techniques have been applied to disentangle functional and structural brain connectivity maps and their pathologic correlate in ALS. Mathematical models applied to diffusion tensor MRI (DT MRI) and resting-state fMRI (RS fMRI) allow to organize the brain into nodes and edges, which interact to form the structural and the functional brain connectome, respectively. The development of predictive models of pathology progression taking into account MRI quantitative features is a “hot topic” for research in the neuroimaging field. The paradigm of the brain structural and functional connectome is an ideal tool to prove the prognostic implications of the network-based degeneration hypothesis, which implied pathologic spreading across either structural or functional interlinked regions, ultimately leading to progression of neurodegeneration.^8^

The aim of this study was to explore the rearrangements of structural and functional connectivity within and among brain networks underlying the clinical stages of ALS, to suggest objective, reproducible measures mirroring disease spreading.

## Methods

### Standard Protocol Approvals, Registrations, and Patient Consents

Local ethical standards committee on human experimentation approved the study protocol, and all participants provided written informed consent.

### Participants

**Figure 1:**
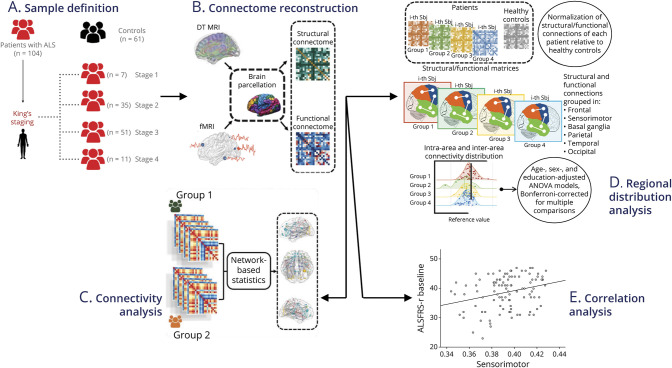
Graphical Representation of Methods (A) Sample division. Patients were stratified according to King's staging system into stages 1, 2, 3, and 4. (B) Structural and functional connectome reconstruction into 220 regions. (C) Connectivity analysis using network-based statistics. (D) Regional distribution analysis. All connections per each patient were normalized relative to controls and grouped into 6 macroregions. (E) Correlation analysis. ALS = amyotrophic lateral sclerosis; ALSFRS-r = ALS Functional Rating Scale-revised; ANOVA = analysis of variance; DT MRI = diffusion tensor MRI.

One hundred forty-five patients with a definite, probable, or probable laboratory-supported diagnosis of ALS according to the revised El Escorial criteria^9^ were consecutively recruited at San Raffaele Institute in Milan between 2010 and 2016. Patients underwent neurologic examination, brain MRI, cognitive screening, and genetic testing at study entry. Thirty-four patients carrying ALS-related pathogenic variants (i.e., 19 *C9orf72*, 8 *TARDBP*, 6 *SOD1*, and 1 *FUS*) and 7 patients with a co-occurrent diagnosis of behavioral variant of frontotemporal dementia (bvFTD)^10^ were excluded. Therefore, we selected a cohort of 104 patients with nondemented ALS who did not carry any known genetic pathogenic variant to be included in this study (Table 1, Figure 1A). At study entry, all patients were receiving treatment with riluzole. Sixty-one age-matched and sex-matched healthy controls (HCs) were also recruited by word of mouth, based on the following criteria: unremarkable neurologic assessment, Mini-Mental State Examination (MMSE) score ≥28, and absence of neurodegenerative diseases in the family history. For this study, all participants showing any of the following were excluded: medical illnesses or substance abuse potentially interfering with cognitive functioning; any additional major systemic, neurologic, or psychiatric conditions; and additional causes of focal or extensive brain damage, such as lacunae and diffuse cerebrovascular disorders at MRI.

**Table 1:** Demographic and Clinical Features of Patients With ALS and HCs

	HC	ALS King's stage 1	ALS King's stage 2	ALS King's stage 3	ALS King's stage 4	*p* Value
N	61	7	35	51	11	—
Age at MRI, y	63.0 ± 8.5	63.7 ± 12.0	67.4 ± 8.1	61.9 ± 11.9	59.8 ± 7.5	0.09
Sex (male/female)	25/36	1/6^a,b^	24/11	27/24	5/6	0.03^d^
Education, y	12.9 ± 4.7^b^	9.1 ± 2.9	10.7 ± 5.0	10.2 ± 4.1	9.4 ± 3.9	0.01^d^
Disease duration, mo	—	12.3 ± 4.2	21.5 ± 24.8	21.8 ± 18.9	19.6 ± 18.2	0.71
ALSFRS-r score (0–48)	—	42.7 ± 2.5	40.8 ± 5.4	37.5 ± 5.1^c^	34.9 ± 6.5^c^	0.001^d^
MRC global score (0–120)	—	114.0 ± 7.7	104.5 ± 17.3	99.6 ± 15.7^c^	95.7 ± 18.2^c^	0.03^d^
Disease progression rate (/month)	—	0.4 ± 0.1	0.5 ± 0.5	0.8 ± 0.6	0.9 ± 0.7	0.17
UMN burden (0–16)	—	11.3 ± 3.1	8.9 ± 4.5	11.5 ± 4.2	12.4 ± 1.7	0.18
Site of onset (limb/bulbar)	—	2/5^a,b^	29/6	39/12	6/5	0.01^d^

Abbreviations: ALS = amyotrophic lateral sclerosis; ALSFRS-r = ALS Functional Rating Scale-revised; HC = healthy control; MRC = Medical Research Council; UMN = upper motor neuron.

Numbers are mean ± SD. *p* Values refer to Pearson χ^2^ test or analysis of variance models, followed by post hoc pairwise comparisons, Bonferroni-corrected for multiple comparisons. Comparisons between clinical variables were adjusted for age and sex.

a Statistically significant difference from ALS stage 2.

b Statistically significant from ALS stage 3.

c Statistically significant difference from ALS stage 1.

d *p* Values <0.05 were considered significant.

### Clinical Evaluation

Clinical evaluation was performed by experienced neurologists blinded to MRI results. Disease severity was assessed using the ALS Functional Rating Scale-revised (ALSFRS-r).^3^ The rate of disease progression was defined as (48 − ALSFRS-r score)/time from symptom onset. Manual muscle testing was assessed based on the Medical Research Council (MRC) scale, and clinical UMN involvement was graded by totaling the number of pathologic UMN signs.^11^ Clinical staging was defined according to the King's College staging system,^5^ therefore assigning patients to stages 1–3 based on the number of body regions (bulbar, cervical, thoracic, or lumbar) showing any sign of clinical involvement or stage 4 in case of nutritional or respiratory failure, that is, requirement for gastrostomy or noninvasive ventilation.

### Genetic Analysis

Blood samples were obtained from 133 screened patients. A repeat-primed PCR assay was used to assess the presence of GGGGCC hexanucleotide expansion in the first intron of the *C9orf72* gene.^12^ A threshold of ≥30 repeats with a typical saw-tooth pattern was considered as pathologic. Optimized PCR protocols were used to amplify the coding sequences and intron/exon boundaries of the *TARDBP*, *SOD1*, and *FUS* genes, looking for known pathogenic variants.^13^ Patients carrying a pathogenic variant in any of these genes were excluded from this study.

### Neuropsychological Evaluation

Ninety-two patients underwent a comprehensive cognitive and behavioral assessment performed by a trained neuropsychologist unaware of MRI results, evaluating global cognitive functioning, reasoning and executive functions, verbal memory, language, mood disturbances, and behavioral alterations.^14^ Details of the neuropsychological evaluation are reported in eAppendix 1 (links.lww.com/WNL/D275).

According to the revised Strong criteria,^15^ patients were classified as showing pure motor impairment (ALS-cn) or displaying cognitive and/or behavioral deficits (ALS-ci/bi). A diagnosis of bvFTD was made according to the established clinical criteria.^10^ Patients who met bvFTD criteria were excluded from this study.

### MRI Acquisition

All participants underwent a brain MRI scan on a 3.0 Tesla Philips Intera scanner. The following sequences were acquired: T2-weighted spin echo, fluid-attenuated inversion recovery, 3D T1-weighted fast field echo, DT MRI, and RS fMRI.^14^ The acquisition protocol is presented in detail in eTable 1 (links.lww.com/WNL/D275). Experienced observers blinded to participants' identity performed the MRI analysis.

### Connectome Reconstruction

An optimized pipeline for brain parcellation, preprocessing of DT MRI and RS fMRI data, and reconstruction of structural and functional brain connectome was used.^14,16,17^

As presented in Figure 1B, cortical and subcortical gray matter (GM) of the brain was parcellated into 220 similarly sized regions (eTable 2, links.lww.com/WNL/D275), to be used as nodes in a graph theoretical approach. Structural and functional connections between each pair of nodes were considered as edges, in fractional anisotropy (FA) for structural connectivity or Pearson correlation coefficients between each pair of nodes for functional connectivity. After reconstructing the structural macroscale connectome for each participant, the structural connectome of an independent HC group was applied as a comprehensive brain connection mask.^17^ Then, each participant's masked structural connectome was used as a mask for the respective functional connectome, to investigate the functional alterations only where structural connections exist, facilitating the biological interpretation of the results.^18^

### Statistical Analysis

#### Clinical and Cognitive Data

Assumption of normal distribution was checked using Q-Q plot, Shapiro-Wilk, and Kolmogorov-Smirnov tests. Demographic and clinical data were compared between groups using Pearson χ^2^ or analysis of covariance (ANCOVA) models adjusted for age and sex, followed by post hoc pairwise comparisons, Bonferroni-corrected for multiple comparisons. ANCOVA models adjusted for age, sex, and education levels were also used to compare cognitive data between groups, followed by post hoc pairwise comparisons, Bonferroni-corrected for multiple comparisons. The significance threshold was set at *p* < 0.05. SPSS Statistics 26.0 software was used.

#### Connectivity Analysis

Network-based statistics (NBS)^19^ was performed to assess structural and functional connectivity between each pair of nodes at a level of significance *p* < 0.05, performing all possible combinations of comparisons between study groups (Figure 1C). We identified the largest (or principal) connected component and the smaller clusters of altered connections.^17,19^ For each contrast, a corrected *p* value was calculated by means of a permutation analysis (10,000 permutations) adjusted for age, sex, and education levels.

#### Regional Distribution Analysis

To assess the distribution of connectivity alterations across ALS stages, the structural/functional connectivity values of each connection for each patient were normalized relative to controls, as described by the following formula (Figure 1D)^14^:
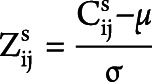
where 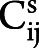 is the connectivity value of the connection between node *i* and *j* for participant s; μ is the mean connectivity value of the considered connection in HCs; and σ is the SD of the connectivity value of such connection in HCs. Subsequently, the 220 regions from both hemispheres were grouped into 6 anatomical macroregions: sensorimotor, parietal, temporal, frontoinsular, occipital, and basal ganglia (eTable 2, links.lww.com/WNL/D275). For each ALS King's stage (1–4), the mean values of intra-area and interarea connectivity were calculated averaging the normalized structural/functional connections belonging to a region (intra) or linking 2 distinct regions (inter), respectively. Finally, ANCOVA models adjusted for age, sex, and education levels were used to compare the intraregional and inter-regional connectivity values between patient groups, followed by post hoc pairwise comparisons, Bonferroni-corrected for multiple comparisons (*p* < 0.05) in SPSS Statistics 26.0.

#### Correlation Analysis

Finally, we tested partial correlations between clinical/cognitive variables and MRI measures showing significant differences between patients and controls using Pearson correlation coefficient (*r*), at the significance threshold of *p* < 0.05, adjusting for age, sex, and—for cognitive variables—education (Figure 1E).

### Data Availability

The data set and codes used for this study will be made available by the corresponding author on request.

## Results

### Demographic and Clinical Data

Demographic and clinical data of patients with ALS are summarized in Table 1. Based on clinical characteristics and number of body regions involved, 7 patients with ALS were categorized into King's stage 1, 35 in stage 2, 51 in stage 3, and 11 in stage 4. Of note, all patients with ALS classified into King's stage 4—that is, meeting criteria for required gastrostomy or noninvasive ventilation—also showed clinical involvement of upper and lower limbs, displaying the involvement of at least 3 body regions (n = 4 with 3 body regions and n = 7 with all 4 body regions involved). Patient groups were comparable for age, sex, and education, except for the ALS stage 1 group showing an overrepresentation of female patients (6/7). ALS patient groups did not differ in disease duration, UMN burden, and ALSFRS-r rate of decline, whereas ALSFRS-r and MRC global scores progressively decreased from stage 1 to stage 4, with ALS stages 3 and 4 patients showing a significant difference of these measures, compared with stage 1. A bulbar presentation was significantly more common in ALS stage 1 patients compared with stages 2 and 3.

### Cognitive and Behavioral Data

Table 2 summarizes neuropsychological features of included patients. Among those who underwent a comprehensive cognitive assessment (n = 85), 61 (72%) were classified as ALS-cn and 24 (28%) as ALS-ci/bi. The relative frequency of cognitive and/or behavioral impairment did not differ across ALS stages (*p* = 0.76). ALS stage 3 patients performed worse than HCs in MMSE and some executive and verbal memory tasks. ALS stage 4 patients performed significantly worse than HCs as regards Wisconsin Card Sorting Test global scores and phonemic fluency indexes. No significant differences of neuropsychological scores were detected between patient groups.

**Table 2:** Neuropsychological and Behavioral Features of Patients With ALS and HCs

	HC	ALS King's stage 1	ALS King's stage 2	ALS King's stage 3	ALS King's stage 4	*p* Value
N	61	5	30	42	8	
Cognitive diagnosis (ALS-cn/ALS-ci/bi)	—	3/2	24/6	28/14	6/2	0.76
MMSE	29.3 ± 0.9	28.2 ± 1.8	28.1 ± 2.6	27.8 ± 2.0^a^	27.5 ± 1.1	0.02^c^
Reasoning and executive functions						
Raven colored progressive matrices	30.9 ± 3.4	26.7 ± 5.4	29.7 ± 3.8	27.1 ± 5.6^a^	28.4 ± 3.8	0.04^c^
Digit span, backward	4.6 ± 1.1	4.5 ± 0.6	3.9 ± 1.2	3.6 ± 0.9^a^	4.0 ± 0.0	0.01^c^
CET	—	14.3 ± 2.9	12.4 ± 3.7	14.9 ± 4.4	16.2 ± 4.1	0.26
WCST, global score	32.8 ± 22.6	71.0 ± 34.5	48.3 ± 39.7	58.9 ± 35.8	106.8 ± 26.0^a^	0.003^c^
Weigl Sorting test	—	11.2 ± 1.5	12.3 ± 2.5	10.9 ± 3.5	11.4 ± 3.6	0.51
Fluency						
Phonemic fluency index	4.7 ± 2.1	4.2 ± 1.6	6.8 ± 3.6	6.9 ± 3.9	11.1 ± 11.9^a,b^	0.01^c^
Semantic fluency index	3.8 ± 0.9	4.8 ± 2.3	4.7 ± 2.3	5.3 ± 3.8	5.9 ± 2.7	0.14
Verbal memory						
Digit span, forward	5.9 ± 0.9	5.0 ± 0.8	5.4 ± 0.9	5.2 ± 1.1^a^	5.0 ± 1.4	0.006^c^
RAVLT, immediate recall	46.4 ± 9.0	41.2 ± 8.4	42.1 ± 12.3	40.0 ± 11.0	41.8 ± 12.1	0.79
RAVLT, delayed recall	9.2 ± 3.3	8.0 ± 1.4	8.3 ± 3.1	9.0 ± 3.5	9.0 ± 3.5	0.91
Language						
Oral noun confrontation naming subtest of BADA	29.8 ± 0.5	29.0 ± 1.1	28.9 ± 1.8	28.6 ± 1.3	29.4 ± 0.9	0.09
Oral verb confrontation naming subtest of BADA	27.7 ± 0.6	27.0 ± 1.4	26.6 ± 2.3	26.6 ± 1.8	26.0 ± 1.7	0.15
Behavioral disturbances						
FBI	—	2.2 ± 2.6	2.8 ± 2.5	2.3 ± 2.9	2.0 ± 2.8	0.87
ALS-FTD questionnaire	—	15.5 ± 23.0	12.7 ± 10.7	13.9 ± 9.9	NA	0.85
Depression						
HDRS	—	5.5 ± 4.7	4.6 ± 4.1	5.6 ± 2.9	9.5 ± 3.3	0.15

Abbreviations: ALS = amyotrophic lateral sclerosis; ALS-ci/bi = ALS patients with cognitive and/or behavioral impairment; ALS-cn = ALS patients cognitively normal; BADA = Batteria per l'Analisi dei Deficit Afasici (Battery for Analysis of Aphasic Deficits); CET = Cognitive Estimation Test; FBI = Frontal Behavioral Inventory; FTD = frontotemporal dementia; HC = healthy control; HDRS = Hamilton Depression Rating Scale; MMSE = Mini-Mental State Examination; RAVLT = Rey Auditory Verbal Learning Test; WCST = Wisconsin Card Sorting Test.

Values are raw scores, reported as mean ± SD. *p* Values refer to Pearson χ^2^ test or analysis of variance models, followed by post hoc pairwise comparisons, Bonferroni-corrected for multiple comparisons. Comparisons between cognitive variables were adjusted for age, sex, and education.

a Statistically significant difference from HCs.

b Statistically significant difference from ALS stage 1.

c *p* Values <0.05 were considered significant.

### MRI Analysis

#### Connectivity Analysis

**Figure 2:**
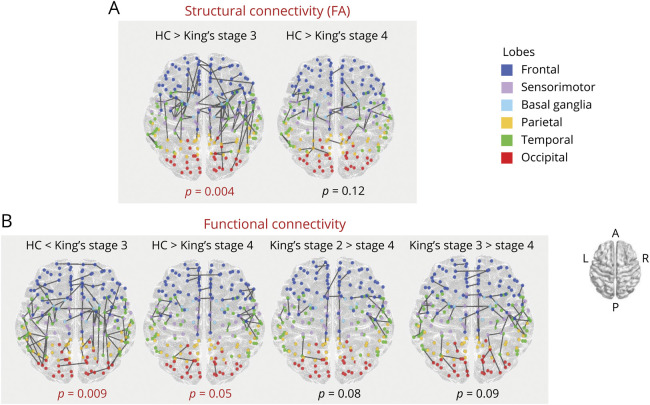
Connectivity Analysis Alterations in structural and functional connectivity in ALS patients stages relative to HCs and each other. Altered structural (top) and functional (down) connections are represented per each significant contrast, respectively (*p* < 0.05). The comparisons were adjusted for age, sex, and education. The node color represents its belonging to specific macroareas (frontal, sensorimotor, basal ganglia, parietal, temporal, and occipital). A = anterior; ALS = amyotrophic lateral sclerosis; HC = healthy control; L = left; P = posterior; R = right.

Compared with HCs, ALS stage 3 patients showed a strong significant reduction (*p* = 0.004) of structural connectivity, mostly involving bilateral, intrahemispheric connections between the sensorimotor, frontal, temporal, and basal ganglia nodes (Figure 2A). ALS stage 3 patients also showed decreased structural connectivity within long-range intrahemispheric connections to parietal and occipital nodes, mostly in the right hemisphere, as well as some anterior frontal and sensorimotor interhemispheric connections. A similar pattern of decreased structural connectivity across sensorimotor, frontal, temporal, and parietal regions was observed in the smaller group of ALS stage 4 patients compared with HCs, although this contrast did not reach statistical significance (*p* = 0.12). No other significant differences of structural connectivity were found between study groups.

Compared with HCs, divergent patterns of functional connectivity were found in ALS stage 3 and ALS stage 4 patients (Figure 2B). In fact, ALS stage 3 patients showed a strong significant increase of functional connectivity (*p* = 0.009) within a vast range of frontal, temporal, sensorimotor, and basal ganglia nodes, with a milder involvement of parieto-occipital connections. Connections showing increased functional connectivity were mostly intrahemispheric, although some interhemispheric connections between frontal, basal ganglia, and occipital regions were also affected. By contrast, ALS stage 4 patients showed a significant decrease of functional connectivity (*p* = 0.05) mostly within frontal, temporal, and sensorimotor regions, with a milder involvement of occipital connections. A similar pattern of decreased functional connectivity in ALS stage 4 was maintained, when compared with ALS stages 2 and 3, although these comparisons did not reach statistical significance (*p* = 0.08 and *p* = 0.09, respectively). No other significant differences of functional connectivity were found between study groups.

#### Regional Distribution Analysis

**Figure 3:**
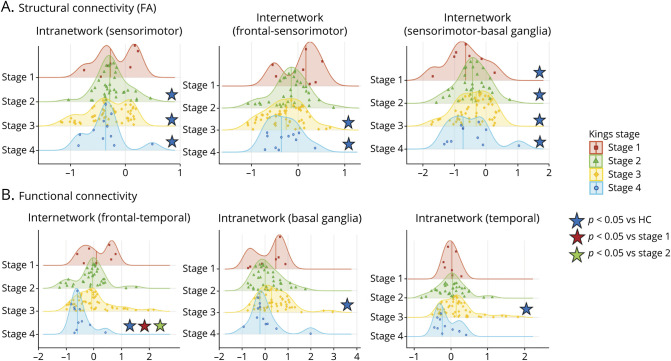
Distribution Analysis of the Structural and Functional Connectivity Damage in Patient Groups The distribution of the structural (A) and functional (B) connectivity damage within and among brain regions is displayed. Distribution curves are normalized relative to control values. The more the curve is shifted toward negative values, the greater is the structural damage. All significant contrasts (*p* < 0.05)—displayed with colored stars—are reported according to age-adjusted, sex-adjusted, and education-adjusted ANOVA models, Bonferroni-corrected for multiple comparisons. ANOVA = analysis of variance; FA = fractional anisotropy; HC = healthy control.

Compared with HCs, patients with ALS of stages 2, 3, and 4 showed significantly decreased intraregional structural connectivity of the sensorimotor regions (*p* = 0.03, *p* < 0.001, and *p* = 0.002, respectively; Figure 3A, eTable 3, links.lww.com/WNL/D275). Decreased inter-regional structural connectivity was also detected for sensorimotor-frontal connections of ALS stages 3 and 4 patients (*p* = 0.03) and sensorimotor-basal ganglia connections of ALS stages 1, 2, 3, and 4 (*p* = 0.04, *p* = 0.01, *p* = 0.001, and *p* = 0.01, respectively), compared with controls. Of note, intrasensorimotor and frontal-sensorimotor structural connectivity showed progressive deterioration at increasing ALS stages (Figure 3A). No other significant differences in intraregional or inter-regional structural connectivity were found between groups.

Inter-regional functional connectivity between frontal and temporal brain regions was significantly decreased in ALS stage 4 patients, as compared with HCs (*p* = 0.03) and ALS stages 1 and 2 (*p* = 0.03 and *p* = 0.05, respectively; Figure 3B, eTable 3, links.lww.com/WNL/D275). ALS stage 4 patients also showed a nearly significant trend (*p* = 0.06) of decreased frontotemporal functional connectivity, compared with ALS stage 3. Compared with HCs, ALS stage 3 patients showed increased intraregional functional connectivity of the basal ganglia (*p* = 0.04) and temporal regions (*p* = 0.05). No other significant differences in intraregional or inter-regional functional connectivity were found between groups.

### Correlation Analysis

**Figure 4:**
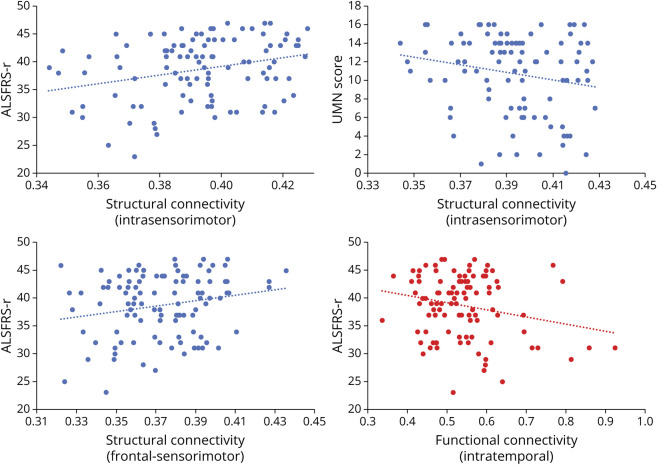
Correlation Analysis Partial correlations between clinical/cognitive and MRI measures (Pearson correlation coefficient R, *p* < 0.05), adjusted for age, sex, and—for cognitive measures—education levels. ALSFRS-r = Amyotrophic Lateral Sclerosis Functional Rating Scale-revised; UMN = upper motor neuron.

Intraregional sensorimotor structural connectivity of patients with ALS was significantly correlated with ALSFRS-r score (*r* = 0.31, *p* < 0.001) and UMN burden (*r* = −0.25, *p* = 0.01) (Figure 4). Inter-regional frontal-sensorimotor structural connectivity also showed significant correlation with ALSFRS-r scores (*r* = 0.24, *p* = 0.02). Intraregional temporal functional connectivity was inversely correlated with ALSFRS-r scores (*r* = −0.22, *p* = 0.03). No significant correlations between MRI and cognitive variables were detected.

## Discussion

This study explored the use of multimodal MRI connectomic techniques to describe the neuroanatomical patterns of brain network involvement in progressive stages of ALS, as described by the King's College staging system. Both whole-brain and regional analyses demonstrated a mostly progressive gradient of decreasing intranetwork and internetwork structural connectivity of sensorimotor regions at increasing ALS stages in our cohort, with earliest involvement of intrasensorimotor and sensorimotor-basal ganglia connections and later damage to frontal-sensorimotor connections (from King's stage 3). Structural MRI connectomic measures showed an evident correlation with patient disability and UMN damage, supporting their ability to mirror disease spreading described by the number of involved body regions. By contrast, functional brain connectivity showed divergent patterns between stages 3 (increase in basal ganglia and temporal circuits, as demonstrated by regional analysis) and 4 (frontotemporal decrease), suggesting a complex interplay between opposite phenomena in late stages of the disease. These findings demonstrate the utility of MRI connectomics to describe pathophysiologic underpinnings of disease progression, stratify patients, and stage brain pathology in ALS.

The most consistent finding shown by the assessment of brain structural connectivity in patients with ALS was the disruption of connections within the sensorimotor network and between sensorimotor, basal ganglia, and frontal regions. The predominant involvement of this motor circuitry interconnecting primary motor, supplementary motor and premotor cortices, and thalamic/basal ganglia nodes is consistent with previous literature,^14,20-22^ supporting the view that a decrease in FA values within these subnetworks might constitute the structural connectomic signature of ALS, in line with proposed neuropathologic and MRI-based disease staging systems describing these regions as the epicenters of TAR DNA–binding protein 43 pathology.^23,24^

Of note, in our study, the structural connectivity values between sensorimotor regions and basal ganglia were significantly reduced across all King's stages, suggesting an early disruption of these connections which would not progress as an increasing number of body regions were involved. Consistent with this hypothesis, increasing evidence provided by previous brain imaging studies in motor neuron disease points toward a significant damage to extraprimary motor regions even in early disease stages.^25,26^ A recent assessment of a network-based model of pathology spread in ALS suggested that the critical “seed” regions to effectively predict regional atrophy distribution might reside not within the primary motor cortex but in the basal ganglia or thalami,^22^ indicating these regions among the earliest sites of pathology accumulation according to this virtual model. By contrast, we demonstrated that the structural connections showing a discrete progression of damage at increasing King's stages included the intrasensorimotor and frontal-sensorimotor networks, which were also those significantly correlating with clinical measures of functional impairment and UMN burden. Based on these observations, we propose the assessment of intrasensorimotor and frontal-sensorimotor structural connectivity as a possible instrumental tool to quantify brain pathology in ALS and allow better definition of clinical stages.

To our knowledge, our study was the first attempting to directly relate the pattern and degree of brain MRI connectomic alterations with the clinical spreading of ALS, as described by the King's staging system. In fact, previous studies testing the use of MRI connectomics to model disease spread in ALS^21,22,27,28^ were essentially in silico simulations evaluating the consistency between network-based diffusion algorithms from a region of interest (e.g., the primary motor cortex) and the distribution of atrophy and/or the postmortem histopathologic staging system proposed by Brettschneider and colleagues.^23^ Such an approach, although incredibly insightful to establish definitive proof for a network-based degeneration hypothesis in ALS, similar to other neurodegenerative conditions,^29^ lacks an immediate utility in a clinical context, where the search for quantitative and reproducible biomarkers of disease staging needs validation from bedside measurements of regional spreading. A few studies have previously tested more conventional neuroimaging approaches to evaluate structural alterations at increasing King's stages in ALS, demonstrating correlations with DT MRI microstructural alterations of the corticospinal tract and body of the corpus callosum,^30^ cervical spinal cord cross-sectional area,^31^ frontotemporal cortical,^32^ and hippocampal and thalamic GM volumes.^33^ However, only some of these reports also showed a concomitant correlation of MRI features with clinical measures of functional impairment.^30,31^ Considering the great prognostic importance of the cumulative number of body regions involved and regional progression time intervals shown by recent epidemiologic studies,^34,35^ the assessment of advanced network-based techniques in our study bridges a gap between the neuroimaging and clinical fields, supporting the use of MRI structural connectomics as a biomarker of progressing neurodegeneration in ALS that might allow a better stratification of patients into upcoming treatment trials.

Compared with structural MRI findings, brain functional data showed a more complex inter-relation with King's stages in patients with ALS, as diverging rearrangements were observed between stages 3 (increased basal ganglia and temporal functional connectivity) and 4 (reduced frontotemporal functional connectivity). The role of functional connectivity modifications in ALS is a long-debated topic in the neuroimaging field, as the current literature includes studies reporting either increased^17,36^ or decreased^37,38^ pattern in patients with ALS. Only a previous longitudinal study demonstrated a mixed picture^39^ of concomitant increased and decreased functional connectivity patterns across different brain networks, similar to our findings in a cross-sectional cohort. Although the design of this study does not allow to draw definitive conclusions, a bell-shaped pattern of evolution might be hypothesized for functional rearrangements in the course of ALS, with a prevalent and progressive increase of functional connectivity in earlier stages of the disease (i.e., King's stages 1–3) and a subsequent decrease as neurodegeneration proceeds toward a terminal stage (i.e., King's stage 4). This interpretation might explain why some previous studies found greater increase of functional connectivity in patients with less severe corticospinal tract damage, preserved motor function,^40^ and shorter disease duration,^41^ whereas others suggested increased functional connectivity as correlating with greater clinical impairment^42,43^ or found no correlation at all.^17^ Heterogeneity among patient populations might lead to variable sampling of patients along the disease course, causing correlations between fMRI and clinical features to be driven by the relative representation of clinical stages in each cohort. For example, this was likely the case for our sample, as a greater prevalence of patients in stage 3 might have driven the significant correlation between increased intratemporal functional connectivity and the degree of clinical impairment. In fact, the same MRI connectomic measure was mostly decreased in ALS stage 4 patients, who showed even lower ALSFRS-r scores compared with stage 3 and co-occurrent significant decrease of frontotemporal functional connectivity. Future longitudinal studies will be able to test our hypothesis, possibly clarifying whether increased intranetwork functional connectivity would represent an early compensatory mechanism eventually “collapsing” when structural integration is completely lost, as a bell-shaped model of evolution might suggest.

Our cohort of patients with ALS—and no comorbid bvFTD, as per the inclusion criteria—did not show any significant differences in cognitive diagnoses across King's stages. This observation rules out a possible influence of cognitive status over MRI connectomic results in this study and is consistent with the lack of different involvement of extramotor networks among stages or any correlation between MRI features and cognitive variables in patients with ALS. Although our findings might also support the view that mild cognitive/behavioral impairment in ALS would represent a distinct phenotype unrelated from the degree of clinical progression, as suggested by a recent connectomic study,^14^ we recognize that they are in contrast with previous studies demonstrating greater risk of cognitive impairment at increasing King's stages^44,45^ and should therefore not be generalized.

A strength of our approach was the inclusion of a relatively homogeneous sample of patients with ALS, who were well-characterized from a cognitive/behavioral point of view, after removing potential biases due to the presence of genetic pathogenic variants or a co-occurrent diagnosis of bvFTD. Moreover, the use of multimodal MRI data allowed to depict the interplay between structural and functional connectivity rearrangements in the same cohort of patients with ALS using a harmonic approach, overcoming most previous studies in this field, which analyzed either DTI or fMRI data.^30-32,40-43^

This study is not without limitations. Some of the ALS staging groups (i.e., stages 1 and 4) were relatively downsized, when compared with other stages. This reflects difficulties in the recruitment of patients who were either very close to symptom onset and, therefore, showed only focal body region involvement or, on the contrary, who were too advanced in the disease course to undergo MRI. Although this imbalance in group sizes calls for extreme caution when interpreting data from stages 1 and 4 patients as regards the possibility of false negatives, the presence of significant findings when assessing these groups asseverates the strength of such results (as in the case of functional connectivity data of stage 4 patients). Moreover, HCs had higher education levels than patients, although the analyses involving neuropsychological and MRI data were adjusted for education. Finally, the cross-sectional design of this study does not allow to draw conclusions regarding the actual evolution of connectivity rearrangements as progressive disease stages are reached by individual patients with ALS overtime.

Notwithstanding these shortcomings, our findings demonstrate the utility of brain MRI connectomics to measure and stage brain pathology in ALS, advocating for the inclusion of measures of structural integrity of the sensorimotor system into the clinical practice, for the definition of prognosis and a correct stratification of patients in the design of pharmacologic trials.

## Appendix Authors

**Table:**

Name	Location	Contribution
Edoardo G. Spinelli, MD	Neuroimaging Research Unit, Division of Neuroscience, and Neurology Unit, IRCCS San Raffaele Scientific Institute; Vita-Salute San Raffaele University, Milan, Italy	Drafting/revision of the manuscript for content, including medical writing for content; major role in the acquisition of data; study concept or design; analysis or interpretation of data
Alma Ghirelli, MD	Neuroimaging Research Unit, Division of Neuroscience, and Neurology Unit, IRCCS San Raffaele Scientific Institute; Vita-Salute San Raffaele University, Milan, Italy	Drafting/revision of the manuscript for content, including medical writing for content; major role in the acquisition of data; analysis or interpretation of data
Silvia Basaia, PhD	Neuroimaging Research Unit, Division of Neuroscience, IRCCS San Raffaele Scientific Institute, Milan, Italy	Drafting/revision of the manuscript for content, including medical writing for content; major role in the acquisition of data; analysis or interpretation of data
Elisa Canu, PhD	Neuroimaging Research Unit, Division of Neuroscience, IRCCS San Raffaele Scientific Institute, Milan, Italy	Drafting/revision of the manuscript for content, including medical writing for content; major role in the acquisition of data; analysis or interpretation of data
Veronica Castelnovo, PhD	Neuroimaging Research Unit, Division of Neuroscience, IRCCS San Raffaele Scientific Institute, Milan, Italy	Drafting/revision of the manuscript for content, including medical writing for content; major role in the acquisition of data; analysis or interpretation of data
Camilla Cividini, PhD	Neuroimaging Research Unit, Division of Neuroscience, IRCCS San Raffaele Scientific Institute, Milan, Italy	Drafting/revision of the manuscript for content, including medical writing for content; analysis or interpretation of data
Tommaso Russo, MD	Neurology Unit, IRCCS San Raffaele Scientific Institute; Vita-Salute San Raffaele University, Milan, Italy	Drafting/revision of the manuscript for content, including medical writing for content; major role in the acquisition of data; analysis or interpretation of data
Paride Schito, MD	Neurology Unit, IRCCS San Raffaele Scientific Institute, Milan, Italy	Drafting/revision of the manuscript for content, including medical writing for content; major role in the acquisition of data; analysis or interpretation of data
Yuri M. Falzone, MD, PhD	Neurology Unit, IRCCS San Raffaele Scientific Institute, Milan, Italy	Drafting/revision of the manuscript for content, including medical writing for content; major role in the acquisition of data; analysis or interpretation of data
Nilo Riva, MD, PhD	Neurorehabilitation Unit, IRCCS San Raffaele Scientific Institute, Milan, Italy	Drafting/revision of the manuscript for content, including medical writing for content; major role in the acquisition of data; analysis or interpretation of data
Massimo Filippi, MD	Neuroimaging Research Unit, Division of Neuroscience, and Neurology Unit, IRCCS San Raffaele Scientific Institute; Vita-Salute San Raffaele University; Neurorehabilitation Unit, and Neurophysiology Service, IRCCS San Raffaele Scientific Institute, Milan, Italy	Drafting/revision of the manuscript for content, including medical writing for content; study concept or design; analysis or interpretation of data
Federica Agosta, MD, PhD	Neuroimaging Research Unit, Division of Neuroscience, and Neurology Unit, IRCCS San Raffaele Scientific Institute; Vita-Salute San Raffaele University, Milan, Italy	Drafting/revision of the manuscript for content, including medical writing for content; study concept or design; analysis or interpretation of data

## Glossary

ALS: amyotrophic lateral sclerosis
ALS-ci/bi: ALS patients with cognitive and/or behavioral impairment
ALS-cn: ALS patients cognitively normal
ALSFRS-r: ALS Functional Rating Scale-revised
ANCOVA: analysis of covariance
bvFTD: behavioral variant of frontotemporal dementia
DT MRI: diffusion tensor MRI
FA: fractional anisotropy
GM: gray matter
HC: healthy control
MMSE: Mini-Mental State Examination
MRC: Medical Research Council
NBS: network-based statistics
RS fMRI: resting-state fMRI
UMN: upper motor neuron
